# An Epitome of Eye, Ear Throat and Nose Diseases

**Published:** 1885-07

**Authors:** 


AN EPITOME OF EYE, EAR, THROAT AND NOSE DISEASES for the Student and Practitioner. By Arthur G. Hobbs, M. D., Professor of Ophthalmalogy, Otology and Laryngology in the Southern Medical College, Atlanta, Georgia. Illustrated with wood cuts and photographs. Philadelphia: Samuel M. Miller. 1885. Atlanta: Jas. F. Lynch. Price $2.50.

The author of this work of over two hundred pages disclaims in the preface any intention of preparing the work as a text-book or reference book for specialists. He states: The work is intended to present in a concise manner the leading points of the subjects of which it treats—those points only that are necessary to the student and general practitioner. He has accomplished his work right well, and has given to those for whom it was intended a very useful book.

